# Effectiveness of psychological interventions to improve quality of life in people with long-term conditions: rapid systematic review of randomised controlled trials

**DOI:** 10.1186/s40359-018-0225-4

**Published:** 2018-03-27

**Authors:** Niall Anderson, Gozde Ozakinci

**Affiliations:** 1 Public Health Department, NHS Borders, Melrose, TD6 9BD UK; 2 School of Medicine, University of St Andrews, St Andrews, KY16 9TF UK

**Keywords:** Long-term, Physical, Conditions, Psychological, Intervention, Health, Quality, Life, Mental, Wellbeing

## Abstract

**Background:**

Long-term conditions may negatively impact multiple aspects of quality of life including physical functioning and mental wellbeing. The rapid systematic review aimed to examine the effectiveness of psychological interventions to improve quality of life in people with long-term conditions to inform future healthcare provision and research.

**Methods:**

EBSCOhost and OVID were used to search four databases (PsychInfo, PBSC, Medline and Embase). Relevant papers were systematically extracted by one researcher using the predefined inclusion/exclusion criteria based on titles, abstracts, and full texts. Randomized controlled trial psychological interventions conducted between 2006 and February 2016 to directly target and assess people with long-term conditions in order to improve quality of life were included. Interventions without long-term condition populations, psychological intervention and/or patient-assessed quality of life were excluded.

**Results:**

From 2223 citations identified, 6 satisfied the inclusion/exclusion criteria. All 6 studies significantly improved at least one quality of life outcome immediately post-intervention. Significant quality of life improvements were maintained at 12-months follow-up in one out of two studies for each of the short- (0–3 months), medium- (3–12 months), and long-term (≥ 12 months) study duration categories.

**Conclusions:**

All 6 psychological intervention studies significantly improved at least one quality of life outcome immediately post-intervention, with three out of six studies maintaining effects up to 12-months post-intervention. Future studies should seek to assess the efficacy of tailored psychological interventions using different formats, durations and facilitators to supplement healthcare provision and practice.

## Background

Long-term conditions (LTC) are complex physical health issues that last a year or longer and require ongoing care and support [1]. As LTC may be treated but not reversed, long-term care for patients and specialised rehabilitation training for staff is required to deal with the permanent and/or disabling nature of conditions [1, 2]. As a consequence of increased exposure to risk factors, the likelihood of experiencing a LTC shows a linear increase with age, with those aged 75 years or older being up to five times more likely to experience a LTC than any other age group [1, 3, 4]. As the proportion of those aged 65 years or older in Europe is projected to increase from 15% in 2000 to 23.5% in 2030, a major and increasing challenge is faced by public health to not only target LTC symptoms, but also the associated increased rates of disability and reductions in both healthy and overall life expectancy [5, 6]. Furthermore, due to LTC resulting from a combination of genetic, physiological, psychological and socio-economic factors, LTC are also becoming increasingly prevalent in younger populations [6].

LTC encompass a wide range of conditions which impact upon one’s physical, psychological, and social functioning. However, as individual LTC may differ in aetiology, presentation and consequence, there is significant variability in the degree to which each LTC is medically understood, diagnosed and treated [1, 6, 7]. For example, cardiovascular disease and diabetes mellitus are two of the most prevalent and increasingly occurring LTC worldwide, and are associated with increased rates of long-term disability, dependency on others for everyday functioning, and depression [6, 8–10]. Chronic obstructive pulmonary disease and dementia are prevalent but under-diagnosed LTC as symptoms may often be mistakenly attributed to an anticipated gradual age-related decline in functioning. However, both conditions relate to increased medical admissions, distressing symptoms, mortality, and disability [6, 11–13]. Medically unexplained physical symptoms (MUPS) – such as chronic fatigue syndrome, irritable bowel syndrome and fibromyalgia – are also LTC that (despite having unknown aetiologies) profoundly impact psychological, emotional and physical functioning, as well as healthcare costs and requirements [14–16]. Furthermore, aforementioned conditions only provide a snapshot of overall LTC types, and disorder-related fatalities are also predicted to increase for manageable conditions such as asthma without further public health intervention [6].

While it is important to understand the causes, presentations, and consequences of LTC in isolation, to effectively understand the burden of LTC it is critical to look at how multiple LTC may co-occur and interact. While the terms ‘*Multi-morbidity*’ and ‘*Co-morbidity*’ are often used interchangeably, the former refers to several LTC coexisting, while the latter refers to multiple disorders stemming from one predominant LTC [17, 18]. Effective determination of the worldwide rates of specific and multi-morbid LTC is complex because of issues with insufficient or inappropriate health measures and analyses being used, and between-country differences in LTC definitions and inclusion criteria [19, 20]. However, regardless of the figures assessed, LTC pose a key challenge as 14–29% of the European population report one LTC and 7–18% report two or more conditions [21]. Furthermore, these conservative estimates consider a limited range of conditions, and when a broader range of LTC is considered these figures may be considerably higher. For example, 27% of 75–84 year olds in Scotland experience two or more LTC [1]. Hence, policy and interventions must not only target specific LTC, but also account for the often multi-morbid nature of LTC.

Health status is an effective measure of healthcare and intervention effectiveness; however, using solely population-level mortality and morbidity rates may be problematic as they only provide a snapshot of effects [22]. As a consequence, subjective measures such as quality of life (QOL), health-related QOL (HR-QOL) and mental wellbeing (MWB) are increasingly being used in healthcare research to assess subjective health status and condition-related burden and coping [22]. QOL is a multi-dimensional concept that includes subjective evaluations of one’s physical, psychological, emotional, social, functional and/or environmental state. Due to the wide range of potential constructs, QOL may be assessed using uni-dimensional, multi-dimensional, and individual measures [23–33]. HR-QOL and MWB are sub-domains of QOL that may be assessed using general or specific measures [23, 34–43]. HR-QOL relates to one’s perception of physical and mental health and may provide a valuable insight into symptomology–psychology links, while MWB relates to one’s ability to cope with life stressors and maintain a healthy mental state which may provide an insight into illness and coping perceptions [23, 34–43].

LTC diagnosis, treatment, and outcomes not only have a significant impact upon patients’ physical functioning, but may also have profound consequences for psychological wellbeing and QOL through affecting emotional, physiological and MWB. This may consequently impact upon medical outcomes through treatment choice and the likelihood of LTC relapse and survival [44–50]. Co-morbid mental health disorders are a key issue in LTC populations [11], with LTC patients being significantly more likely to be diagnosed with depressive and/or anxiety disorders [51, 52]. This may relate to poorer health outcomes and self-care, more severe symptoms, reduced medical adherence, and increased unhealthy behaviours, healthcare spending, and disorder-related death rates [51, 52]. Despite this, traditional medical models often overlook key psychological variables through employing a paternalistic care approach where clinicians exercise predominant authority over patients’ care [53–55]. Therefore, as LTC outcomes not only relate to healthcare treatment but are also intrinsically linked to psychological wellbeing and mental health, the provision of psychological interventions and therapies is critical for LTC healthcare services and patient outcomes [11, 56, 57].

Previous systematic reviews (SR) have demonstrated efficacy for psychological interventions (provided in a wide range of formats) to improve both QOL and physical health outcomes in specific LTC patients. For example, mindfulness for multiple sclerosis and cancer, psychosocial interventions for diabetes and cancer, cognitive behavioural therapy (CBT) and relaxation for recurrent headaches, and internet-based CBT or coaching for chronic somatic conditions [58–66]. However, to the researchers’ knowledge, there has not previously been a SR that attempts to only assess studies with high scientific rigour that utilise psychological interventions across LTC in order to provide valid comparisons for the effectiveness of interventions and guide LTC healthcare development. As aforementioned, as research has demonstrated that LTC may have profound physiological and psychological effects [1, 6, 8–16], rates of specific and multi-morbid LTC are high and predicted to rise [3–6, 17, 18, 21], and psychological interventions may improve both QOL and physical functioning [56–66], it is crucial to determine which interventions may be effective across conditions.

The rapid SR aimed to examine the effectiveness of a variety of psychological interventions that seek to improve generic or specific QOL, HR-QOL and/or MWB in people with LTC to determine whether specific interventions may be viable and efficacious for general LTC healthcare implementation. As randomised controlled trial (RCT) designs are the most rigorous and effective method for determining whether intervention–outcome relationships are present [67], and to ensure valid comparisons were possible between studies, only RCTs with a usual care control (UCC) condition which directly target and assess patients with a current LTC diagnosis were included. To ensure the review assessed the most up-to-date research, only studies published between 2006 and February 2016 were included. Furthermore, despite a general dose and duration effect being present for psychological intervention effectiveness, evidence relating to the optimum duration of psychological interventions for LTC to achieve maximum effectiveness is mixed [62, 68, 69]. Therefore, an *ante hoc* decision was taken to categorise studies by intervention facilitation duration, encompassing short- (0–3 months), medium- (3–12 months) and long-term (≥12 months) study classifications.

## Methods

### Rapid systematic review

Rapid SR are a form of streamlined SR that may be used by healthcare professionals to guide policy in a time-frame that may not be possible using traditional SR methods. While they do not provide as in-depth information and should not be viewed as a substitute for traditional SRs, rapid SR may have important implications for healthcare decision-making through using systematic methods to provide high-quality information and draw significantly similar conclusions to a traditional SR [70–72]. As the review was conducted during NHS employment and aimed to influence healthcare policy, utilizing a SR procedure was deemed the most feasible and practical approach based on two key considerations. First, in order for the research to have implications (not only for research but also) for healthcare, it was critical that high quality information was provided using limited time and resources [70]. Second, as the research was conducted during NA’s NHS employment as one competency of a two-year professional doctorate-level Health Psychology qualification, the ability to generate a complete draft of findings for NHS stakeholders within a maximum of 6 months (as opposed to up to 2 years for a traditional SR) [70–72] was deemed the most appropriate approach. Therefore, two researchers (NA, GO) followed traditional SR procedures but without searching grey literature and with only one researcher (NA) involved until data extraction was completed. The implications of adopting this approach are presented in *‘Rapid Systematic Review Strengths and Limitations’*.

### Search strategy, selection criteria and data extraction

Searches were conducted on 19.02.2016 by one researcher (NA) using EBSCOhost to access PsychInfo (1967–2016) and PBSC (1974–2016), and OVID to access Medline (1946–2016) and Embase (1974–2016). Both databases were searched using key terms (Table 1), with potential citations suitability assessed using the pre-defined inclusion/exclusion criteria (Table 2). Due to the multi-dimensional nature of QOL there is currently no universally accepted definition of QOL [22, 25]. Therefore, an *ante hoc* decision was made to manually assess individual studies for the presence or absence of QOL rather than include it in the search terms. Additionally, only RCTs with a UCC were included in order to ensure that valid comparisons of rigour and effectiveness were possible between different interventions and LTC [67]. Data were extracted using a template developed from the COCHRANE criteria [73]. As the SR aimed to guide public health policy, the Effective Public Health Practice Project (EPHPP) ‘*Quality Assessment Tool for Quantitative Studies*’ was used to assess study quality [74].

**Table 1:** Database Search Terms

Stage	Criteria	EBSCO	OVID	Combined
1	(psych* AND interven*)	242,630	333,035	575,665
2	AND ((long* AND term* AND physical* AND condition*) OR ((persist* AND physical* AND health) AND (issue* OR problem*)))	793	1430	2223

**Table 2:** Review Selection Criteria

Component	Inclusion	Exclusion
Population	Any LTC (including MUPS) not limited to the conditions discussed in the introduction e.g. kidney or inflammatory bowel disease	Mental health or psychiatric conditions in the absence of LTC
	Any age group from school-aged adolescents (≥ 10 years) onwards in order to ensure appropriate levels of understanding and communication of QOL domains	Pre-school or primary school children (0–9 years)
	Any gender	No gender exclusions
	Any cultural, education or socio-economic status	No cultural, education or socio-economic exclusions
	Any care setting or delivery format	No care setting or delivery format exclusions
Intervention	Psychological intervention (in any format) including those which include alternative but related terminology e.g. cognitive behavioural therapy (CBT) or mindfulness	Non-psychological interventions
	Target and assess LTC patients directly	Psychological interventions designed to indirectly target LTC patients (through clinicians, family, carers etc.)
	Any facilitator	No facilitator exclusions
Study Design	RCT (Level I Quantitative evidence)	Levels II-V Quantitative evidence, qualitative studies, book chapters, dissertations, SR and meta-analysis papers, unpublished journals or grey material
	Journal articles published in English	Non-English publications
	Comparisons made between intervention and UCC at all relevant points	No intervention and/or UCC conditions
	Published between 2006 and February 2016	Published prior to 2006 and after February 2016
Outcomes	QOL, HR-QOL and/or WMB	Non-psychological assessment
	Assess patients directly	Measures that indirectly assess patients (through clinicians, family, carers etc.)

## Results

### Study selection

**Fig. 1:**
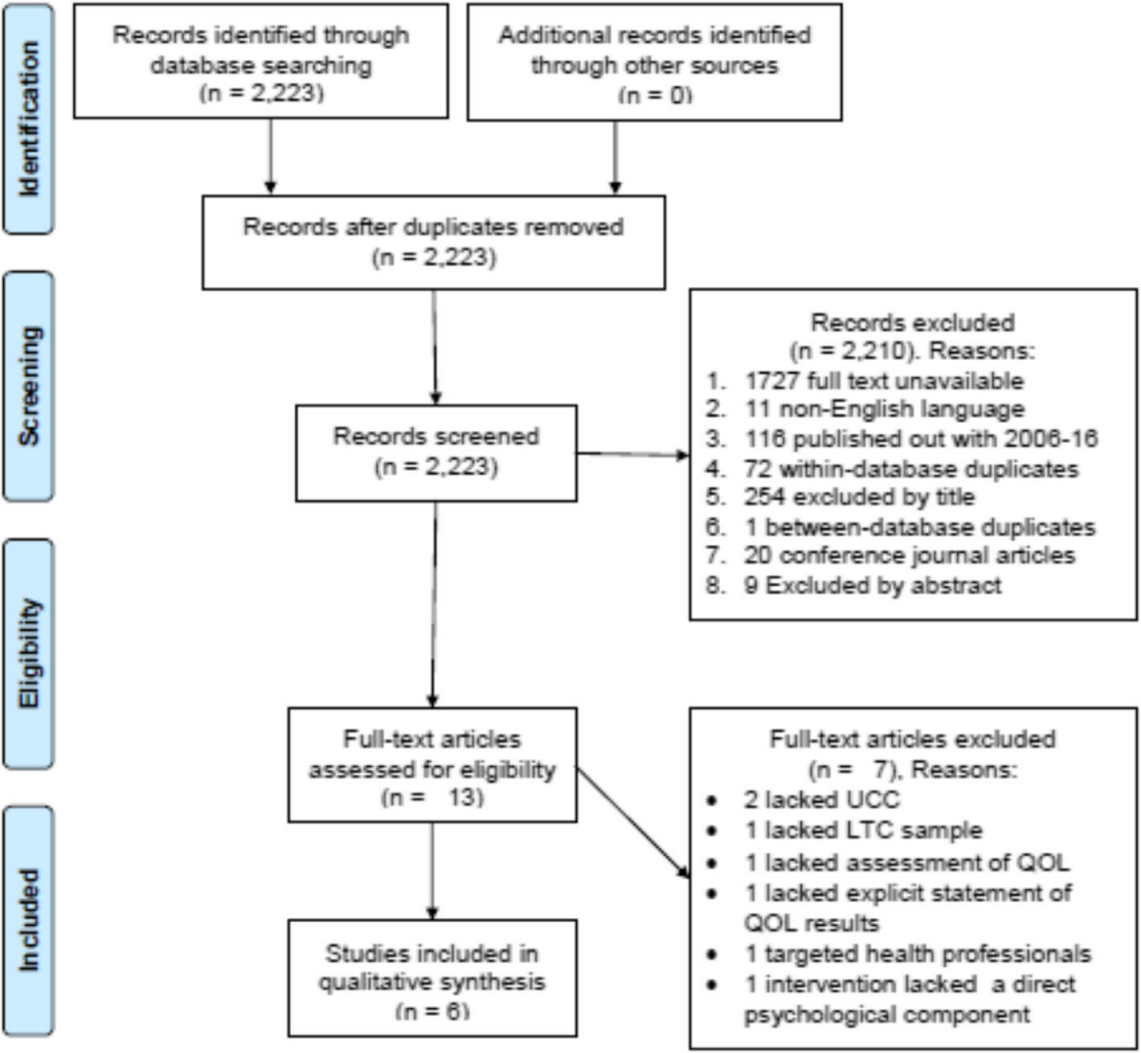
Study Selection Process

The PRISMA flowchart (Fig. 1) demonstrates the process used to narrow 2224 prospective citations to 13 studies based on titles and abstracts [75–87], with 6 studies satisfying the inclusion/exclusion criteria based on full articles [82–87].

### Study characteristics

Key study features, measures, results (including significance values and effect sizes where stated), and authors’ conclusions from the 6 eligible studies are presented in Table 3. The six studies [82–87] encompass a variety of psychological interventions and durations: 2 were short-term (0–3 months) [82, 85], 2 were medium-term (3–12 months) [84, 86], and 2 were long-term studies (≥12 months) [83, 87]. Facilitators of the interventions varied considerably between studies, with nurses facilitating 3 interventions [83, 85, 87], and the remaining 3 studies being facilitated by health educators [82], CBT therapists [84], and clinical psychologists [86]. Additionally, each intervention focussed on a different LTC; comprising asthma [82], human immunodeficiency virus (HIV) [83], MUPS [84], congestive heart failure (CHF) [85], knee osteoarthritis [86], and head & neck cancer (HNC) patients [87]. Five studies compared a UCC with one intervention [82–85, 87], while one study contrasted multiple interventions with a UCC [86]. Furthermore, all 6 studies comprised samples of both genders aged 18 years or over, and assessed (among other measures) generic and/or specific measures of QOL, HR-QOL and/or MWB [82–87].

**Table 3:** Study Characteristics

Author & Location	Participant Demographics	Intervention Length, Content & Groups	Measures & Follow-up	Reported Results	Authors’ Conclusions
Baptist et al. [82]; Nether-lands (*RCT*)	*N* = 70, female 77%, ≥65 years, asthma. Attrition 10%	6-week health educator-led self-regulation intervention; two conditions: 1. Self-regulation training (i) 3-weekly health education group sessions (ii) 3-weekly one-on-one telephone sessions 2. UCC	Measured 0, 1, 6 and 12 months post-intervention. Assessed on: 1. Asthma-related QOL (MAQLQ), including individual components of activity, emotions, environment and symptoms 2. Asthma-related control (ACQ) 3. Hospitalisations 4. Emergency department visits	Significance Level Employed: *p* ≤ 0.05 [Effect sizes not reported] 1. Significant intervention effects for overall asthma related QOL 1 month (*p < 0.001*), 6 months (*p = 0.031*) and 12 months post-intervention (*p = 0.045*) 2. Significant individual intervention effects: (i) 1 month post-intervention for QOL symptoms* (p = .001)* and environment *(p = 0.001)* (ii) 12 months post-intervention for QOL activity (*p* = 0.04) 3. Significant intervention effects for asthma-related control 1 month (*p = .03*) and 12 months *(p = 0.02),* but not 6 months (*p = 0.21*), post-intervention 4. Significant intervention effect for hospitalisations at 12 (*p = 0.04*), but not 6 months (*p = 0.07*), post-intervention 5. Non-significant intervention effects at all time points for asthma-related QOL emotions (*0.38 ≥ p ≥ 0.07)* and emergency department visits (*0.58 ≥ p ≥ 0.54)*	“By targeting a disease from an individual’s perspective rather than illness from a physician’s perspective, this intervention is ideally suited to improve outcomes in elderly adults.”
Blank et al. [83]; USA (*RCT*)	*N* = 238, female 46%, ≥18 years, HIV. Attrition QOL 25%, biomarker 39%	12 month community-based nurse management of mental and medical conditions; two conditions: 1. Weekly psycho-education and symptom management meeting with a community nurse 2. UCC (PATH+ pathway)	Measured at baseline, 3, 6, 12 (end of intervention) and 24 months (12 post-intervention). Assessed on: 1. Health-related QOL (SF-12), including mental and physical health 2. HIV biomarkers (HIV viral load, CD4)	Significance Level Employed*: p < .05* 1. Model A: viral load and SF-12 mental outcomes 12 months post-intervention (i) Viral load treatment effect on β = − 0.138 (*p < 0.05*) (ii) Mental treatment effect on β = 0.91 (p < 0.05) (iii) Goodness of fit significant: *p = 0.01; RMSEA = 0.055* (0.027, 0.080) 2. Model B: CD4 and SF-12 meant outcomes 12 months post-intervention (i) CD4 treatment effect on β = 0.486 (*p ≥ 0.05*) (ii) Mental treatment effect on β = 0.91 (p < 0.05) (iii) Goodness of fit significant: *p = 0.04; RMSEA = 0.049 (0.018, 0.075)* 3. Model C: viral load and SF-12 physical outcomes 12 months post-intervention (i) Viral load treatment effect on β = − 0.136 (*p < 0.05*) (ii) Physical treatment effect on β = − 0.42 (p ≥ 0.05) (iii) Goodness of fit significant: *p = 0.01; RMSEA = 0.058* (0.082, 0.083) 4. Model D: CD4 and SF-12 physical outcomes 12 months post-intervention (i) CD4 treatment effect on β = 0.485 (*p ≥ 0.05*) (ii) Physical treatment effect on β = − 0.42 (*p* ≥ 0.05) (iii) Goodness of fit non-significant: *p = 0.10; RMSEA = 0.045 (0.009, 0.072)*	“Implementation of community-based nurse disease management for this population and other complex patient populations may have significant impact on viral load, immune functioning, and health-related quality of life.”
Escobar et al. [84]; USA (*RCT*)	*N* = 172; Female 88%, 18–75 years; MUPS. Attrition 45%	3-month cognitive behavioural therapist-led CBT intervention; two conditions: 1. 10 sessions of structured CBT and a consultation letter 2. UCC (and a consultation letter)	Measured pre-intervention (baseline), immediately, and 6 months post-intervention. Assessed on 1. Severity of somatic symptoms (PHQ-15 scale of PRIME-MD) and current somatic symptoms (VAS) 2. Functional status (physical functioning subscale from MOS-10) 3. Anxiety (HAM-A) 4. Depression (HAM-D)	Significant Level Employed: *p* < .05 [Effect sizes not reported] 1. Significant intervention effects: (i) Immediately post-intervention for severity of somatic symptoms (*p = 0.1*), current somatic symptoms (*p = 0.01*) and depression (*p = 0.2*) (ii) 3 months post-intervention for severity of somatic symptoms (*p = 0.03*) 2. Significant group-by-time intervention effect for severity of somatic symptoms (*p = 0.03*) immediately post-intervention 3. The intervention non-significantly affected all other outcomes (*p = unspecified*)	“...with proper training of clinicians, the intervention described herein should be relatively easy to implement in many primary care settings... therefore needs to be considered for future studies as well as for current practice.”
Smeulders et al. [85]; Nether-lands (*RCT*)	*N* = 317, Female 27%, ≥ 18 years; CHF. Attrition 16%	6-week cardiac nurse and peer role model co-facilitated structured self-management programme; two conditions: 1. Weekly 2.5-h structured self-management programme 2. UCC	Measured pre-intervention (baseline) and immediately, 6 months and 12 months post-intervention. Assessed on: 1. Psychosocial attributes, including general self-efficacy (GSES) and cardiac-specific self-efficacy (CSE) 2. Perceived control (PM) 3. Cognitive symptom management (Coping with symptoms scale of ASES) 4. Self-care behaviour (EHFScBS) 5. QOL, including general QOL (RAND-36), cardiac-specific QOL (KCCQ), perceived autonomy (VAS), and anxiety and depression (HADS)	Significance Level Employed: *p* ≤ 0.05 1. Significant intervention effects immediately post-intervention for: (i) Cardiac-specific QOL (*p = 0.005*, *d = 0.06*). (ii) Cognitive symptom management (*p = 0.008, d = 0.34*) (iii) Self-care behaviour (*p = 0.008, d = 0.18*) 2. Non-significant intervention effects were present immediately, 6 months, and 12 months post-intervention for all other measures (*0.986 ≥ p ≥ 0.052*)	“...this programme was considered feasible by both programme leaders and participants... but showed limited, mainly short-term effects...” “More effective alternatives need to be found in nursing care to support self-management behaviour by patients...”
Somers et al. [86]; USA (*RCT*)	*N* = 232, Female 79%, ≥ 18 years; Knee osteoarthritis. Attrition 30%	24-week clinical psychologist-led PCST and BWM programme; 4 conditions: 1. PCST– 12 60-min weekly group sessions followed by 12 biweekly sessions 2. BWM programme – 12 60-min weekly group sessions followed by 12 biweekly sessions 3. Combined PCST/BWM programme 4. UCC	Measured pre-intervention, and immediately, 6 months and 12 months post-intervention. Assessed on: 1. Pain, physical disability and psychological disability (AIMS, WOMAC) 2. Gait velocity (WOMAC) 3. Pain catastrophizing (Catastrophizing scale of CSQ) 4. Self efficacy, including arthritis self-efficacy (ASES) and weight self-efficacy (WEL) 5. Weight (kg) and BMI (kg/m)	Significance Level Employed: p < .05 [Effect sizes not reported] 1. Significant overall treatment effect 12-months post-intervention for: (i) Pain (AIMS: *p = 0.007*; WOMAC: *p = 0.0002*) (ii) Physical disability (AIMS: *p < 0.0001;* WOMAC: *p < 0.0001*) (iii) Stiffness/Gait velocity (*p = 0.0017*) (iv) Pain catastrophizing (*p = 0.02*) (v) Arthritis self-efficacy (*p < 0.0001*) and weight self-efficacy (*p = 0.0002*) (vi) Weight (*p < 0.0001*) and BMI (*p < 0.0001*) 2. The combined PCT/BWM intervention significantly improved outcomes 12-months post-intervention compared with: (i) BWM for pain (AIMS: p = 0.01; WOMAC: *p* = 0.002*),* physical disability (AIMS: *p < 0.0001;* WOMAC: *p < 0.0001*), stiffness/gait velocity (*p = 0.004*), pain catastrophizing (*p = 0.008*), arthritis self-efficacy (*p = 0.0002*), weight self-efficacy (*p = 0.003*), weight (*p = 0.0014*) and BMI (*p = 0.0004*). (ii) PCST for pain (WOMAC: p = 0.01*),* physical disability (AIMS: *p < 0.0001;* WOMAC: *p = 0.0001*), stiffness/gait velocity (*p = 0.02*), psychological disability (*p = 0.*05), arthritis self-efficacy (*p = 0.004*), weight self-efficacy (*p = 0.02*), weight (*p < 0.0001*) and BMI (*p < 0.0001*) (iii) UCC for pain (AIMS: *p = 0.02*; *WOMAC: p = 0.0002),* physical disability (AIMS: *p < 0.0001;* WOMAC: *p = 0.0001*), stiffness/gait velocity (*p = 0.02*) pain catastrophizing (*p = 0.04*), arthritis self-efficacy (*p < 0.0001*), weight self-efficacy (*p = 0.0001*), weight (*p < 0.0001*) and BMI (*p < 0.0001*) 3. The combined PCST/BWM intervention non-significantly affected all other outcomes 12-months post-intervention *(0.98 ≥ p ≥ 0.16)*	“...significant benefits are provided by simultaneously training overweight and obese OA patients to increase the effectiveness of their pain coping skills and manage their weight.” “It may be that PCST gives patients pain coping skills, which enhances their ability to comply with the needed lifestyle changes to lose weight (i.e., increasing activity, decreasing eating).
Van Der Meulen et al. [87] Nether-lands (RCT)	*N* = 205, female 30%, ≥ 18 years; HNC. Attrition 13%	12-month nurse-led counselling intervention for depressive symptoms; two conditions: 1. 6 bimonthly 45–60 min psychosocial sessions (and a regular medical follow-ups) 2. UCC (regular medical follow-ups)	Measured pre-intervention (baseline), and during the intervention at 3, 6 and 9 months. The primary assessment end-point was immediately post-intervention (i.e. 12 months after baseline). Assessed on: 1. Depression (CES-D) 2. Physical symptom related QOL (EORTC QLQ), including pain, swallowing, senses, speech, teeth, opening mouth, dry mouth, sticky saliva, and coughing	Significance Level Employed: *p* ≤. 05 [Exact significance values and effect sizes not reported; between-group change means (CI 95%) listed instead] 1. Significant intervention effect immediately post-intervention for: (i) Depressive symptoms in the overall sample (∆ mean = − 2.8) and depressive sub-group (∆ mean = − 5.2) (ii) Overall physical symptoms in the overall sample (∆ mean = unspecified) and depressive sub-group (∆ mean = unspecified) (iii) Physical symptoms of pain (∆mean = − 9.9), swallowing (∆mean = − 8.0), opening mouth (∆mean = − 14.6) and coughing (∆mean = 10.9) in overall sample (iv) Physical symptom of opening mouth (∆ mean = − 23.2) for the depressive sub-group 2. 2. Non-significant intervention effects were present for all other measures (10.5 ≥ ∆ mean ≥ − 10.6)	“...psychotherapeutic interventions are effective in reducing depressive symptoms in general cancer patients.” “...NUCAI is feasible and effective for reducing depressive symptoms of patients with HNC, particularly for those with raised levels of depression symptoms.”

### Study quality assessment

EPHPP quality assessment [74] involves assessing studies based on 6 key components (Table 4). Each component comprises multiple choice questions for which scores are combined to provide an overall component rating of ‘*Strong*’, ‘*Moderate*’ or ‘*Weak*’. All component ratings are then combined to provide an overall quality rating of ‘*Strong*’ for no ‘*Weak*’ components, ‘*Moderate*’ for one ‘*Weak*’ component, and ‘*Weak*’ for two or more ‘*Weak*’ components.

**Table 4:** EPHPP Quality Assessment

Study	EPHPP Sub-domains						EPHPP Overall Rating
	*Selection Bias*	*Design*	*Confounders*	*Blinding*	*Data Collection*	*Withdrawals & Dropouts*	
Baptist [82]	W	S	S	S	S	S	M
Blank [83]	S	S	S	S	S	W	M
Escobar [84]	S	S	S	M	S	W	M
Smeulders [85]	W	S	S	W	S	S	W
Somers [86]	S	S	S	M	S	M	S
Van Der Meulen [87]	M	S	S	S	S	S	S

Study Quality Rating*: W:* Weak*; M:* Moderate*; S:* Strong

### Short-term interventions (0–3 months)

Two short-term interventions were present. Baptist et al. [82] offered a 6-week health educator-led self-regulation intervention for asthmatic patients (*N* = 70), comprising 3 consecutive weekly health education group sessions followed by 3 weekly one-to-one telephone sessions. Health educators received a 2-day training session on self-regulation and asthma management principles which was used to conduct tailored self-regulation interventions. This involved patients’ self-selecting a specific asthma-related problem that they wished to address before planning how to achieve positive outcomes and cope with potential asthma-related issues. Significant improvements were present 12-months post-intervention for overall asthma-related QOL, activity, control and hospitalisations. QOL symptom and environment improvements were present 1-month post-intervention, and non-significant changes occurred for QOL emotions or emergency department usage.

Smeulders et al. [85] offered a 6-week, 150-min per week structured self-management programme for CHF patients (*n* = 317). The intervention was co-facilitated by a cardiac nurse specialist and a CHF patient (acting as a peer role model) who were both trained on a 4-day ‘*Chronic Disease Self-Management Programme*’ [88] by a research and CHF nurse specialist. This incorporated four strategies to enhance self-efficacy over one’s condition: skills mastery, behaviour modelling, social persuasion and symptom reinterpretation. Significant improvements were present immediately (but not at 6- or 12-months) post-intervention for cardiac-specific QOL, cognitive symptom management and self-care behaviour. However, non-significant intervention effects were present at all time-points for perceived control, general self-efficacy, and all other QOL outcomes (general QOL, perceived autonomy, and anxiety and depression).

### Medium-term interventions (3–12 months)

Two medium-term interventions were present. Escobar et al*.* [84] offered 10, 45–60-min CBT therapist-led sessions over a 3-month period to MUPS patients (*n* = 172). Two therapists received training from two authors employed by Departments of Psychology and Psychiatry respectively, with protocol adherence routinely evaluated using “taped” recordings. Key topics included managing physical distress, relaxation, activity regulation, emotional awareness, cognitive restructuring and interpersonal communication. The intervention significantly improved patient-rated depression and current somatic symptoms, and physician-rated global severity of symptoms, immediately post-intervention. Only changes to patient-rated somatic symptoms were maintained 6-months post-intervention and no effects were present for anxiety or physical functioning.

Somers et al. [86] ‘*Pain Coping Skills Training*’ (PCST) and ‘*Behavioural Weight Management*’ (BWM) co-interventions for knee osteoarthritis patients (*n* = 232) were conducted by clinical psychologists (with 1–6 years experience in their respective area), under the supervision and training of an experienced senior clinical psychologist. The intervention spanned 24 weeks, comprising 12 weekly groups sessions followed by 12 weeks of sessions every second week for the remainder of the intervention. One group received BWM based on the '*LEARN*' programme [89], which focused on lifestyle, exercise, attitudes, relationships and nutrition. The second group received PCST, which focused on maladaptive pain catastrophizing and adaptive coping strategies. The third group received both BWM and PCST programmes. While the study did not utilise a generic measure of QOL, the combined intervention demonstrated significant improvements compared to UCC 12-months post-intervention for arthritis- and weight-specific self-efficacy, pain symptoms and catastrophizing, physical disability and stiffness, weight, and BMI.

### Long-term interventions (≥12 months)

Two long-term interventions were present. Blank et al. [83] offered weekly community-based psycho-education and symptom management sessions (of unspecified duration) over a 12-month period to HIV patients (*n* = 238). Four Advanced Practice Nurses facilitated psycho-education sessions for coping with barriers and self-care, and provided resources to support patients’ to organise their medication regimens. In addition, the Practice Nurses coordinated a multi-disciplinary team of physical and mental healthcare providers to provide tailored medical and mental healthcare. Growth curve analyses were used to assess outcomes, demonstrating significant improvements 12-months post-intervention for the HR-QOL mental health subscale and viral load. However, non-significant improvements were present for the HR-QOL physical health subscale and immune functioning.

Van Der Meulen et al. [87] offered six bimonthly 45-min nurse-led, problem-focused counselling sessions for depressive symptoms to HNC patients (*n* = 205) over a 12-month period. Three experienced oncology nurses received a one-day training course from two psychologists and one investigator on the ‘*Nurse Counselling and After Intervention*’. Session recordings were reviewed every 2 months to assess intervention quality. The intervention focussed on managing the physical, psychological and social consequences of HNC, restructuring illness cognitions and beliefs, education and behavioural relaxation training, and providing emotional support. Significant improvements were present immediately post-intervention (both in the overall sample and depressive subgroup) for the primary endpoint of depressive symptoms and secondary endpoint of overall physical symptoms.

## Discussion

### General statement

The review aimed to examine the effectiveness of psychological interventions to improve specific or generic components of QOL, HR-QOL and/or MWb in people with LTC, with a view to advising LTC healthcare provision. The findings, strengths, limitations and implications of studies, and the strengths and limitations of the current review and rapid SR procedure, are discussed.

### Short-term interventions (0–3 months)

#### Six-week self-regulation for older adult asthmatics

Baptist et al. [82] trained health educators on a two-day programme which enabled them to facilitate a six-week self-regulation intervention. As a consequence of the self-regulation intervention, significant improvements occurred for older adults’ overall asthma-related QOL and control up to 12-months post-intervention. The key hallmarks of the self-regulation approach was to facilitate patients’ self-identification of a specific condition-related issue and potential barriers and goals, in order to provide tailored support and increase patients’ self-efficacy over their condition. This approach has also been used to achieve positive outcomes for heart disease and medical noncompliance in older adults [90, 91]. Therefore, when combined with the low attrition rate (7%) [82] and self-regulation concepts not being unique to asthma [92], self-regulation provides promise as an effective and acceptable form of intervention to improve QOL in older adults. Despite receiving ‘*Strong*’ ratings for all but one quality component, the study received a ‘*Weak*’ ‘*Selection Bias*’ rating due to only 54% of those approached agreeing to participate, which may have two potential implications. First, this may indicate a lack of interest in self-regulation interventions potentially due to this approach differing from anticipated traditional asthma care approaches [82]. Second, while double-blinding improves methodological quality [93], a lack of awareness of intervention procedures and potential benefits may have impact enrolment. Additionally, as highlighted by the authors, the study was limited by using a single site and required a certain threshold of patient communicative ability to contribute to group discussions. Therefore, while additional studies and a cost-benefit analysis would be required to determine the efficacy of larger scale programmes, and consideration is required for the enrolment confounds, the study demonstrated that a short-term, health educator-led self-regulation intervention may have promising implications for LTC healthcare.

#### Six-week structured self-management for CHF

Smeulders et al.’s [85] 6-week structured self-management intervention, co-facilitated by a trained cardiac nurse specialist and a CHF peer role model, significantly improved cardiac-specific QOL immediately post-intervention. However, effects were not maintained at 6- or 12-months follow-up, and no other QOL improvements occurred. Despite having four ‘*Strong*’ components, the study received an overall ‘*Weak*’ EPHPP quality rating due to unspecified ‘*Blinding*’ of patients and clinicians, and a ‘*Selection Bias*’ as only 44% of eligible patients participated. As justification for non-participation varied considerably – from a lack of interest to physical, psychosocial or cognitive problems preventing participation – a qualitative study to further explore enrolment issues may be beneficial to determine whether the intervention was sufficiently tailored to complex CHF needs. While the authors proposed that non-significant effects may have resulted from insufficient intervention length or intensity above the “relatively high level” of Dutch standard care, a similar medium-term (15 weeks) self-management intervention improved physical but not emotional QOL [94]. Therefore, despite positive short-term results, further research is required to understand the mechanisms behind the low participation and lack of long-term QOL effects for structured self-management, with a view to using this to develop and trial more tailored interventions.

#### Overall short-term interventions

Despite both short-term interventions reviewed [82, 85] comprising 6-week programmes, considerable differences were present between-interventions that may have influenced outcomes. First, the self-regulation intervention was solely facilitated by health educators, while the CHF intervention was co-facilitated by a nurse and a patient ‘*peer leader*’. While peer leaders were trained to effectively facilitate the intervention, potential differences in pre-existing knowledge and experience associated with not being a trained healthcare professional may have influenced the content, approach and style of programme adopted, and subsequently QOL outcomes. Second, research into the mechanisms behind why the 2-day (but not the 4-day) training resulted in significant long-term QOL improvement would be beneficial. Three possible explanations for this include potential differences in the quality of training, that health educators may benefit more from short-term training than nurses and/or peer leaders, and/or that additional information provided during the longer training may have resulted in a more structured but less tailored approach being adopted with patients. Third, as asthma and CHF differ considerably in emotional, physical and social outcomes [95, 96], this may have impacted the long-term maintenance of intervention effects post-intervention and consequently QOL outcomes. Fourth, methodological differences may have impacted outcomes due to the discrepancy between Blank et al.’s [82] ‘*Moderate*’ and Smuelders et al.’s [85] ‘*Weak*’ EPHPP quality ratings. However, despite considerable differences, both studies demonstrated that interventions which actively engage and involve the patient in their care may significantly improve at least short-term QOL, and that, while achieving initial buy-in for these types of interventions may be challenging, once enrolled attrition rates were low. Therefore, while cost-benefit analyses and further research are required to determine viability and overcome current limitations, short-term psychological interventions that actively involve patients demonstrated initial promise for improving QOL, with self-regulation demonstrating particular promise.

### Medium-term interventions (3–12 months)

#### Three-month CBT for medically unexplained symptoms

Escobar et al.’s [84] structured CBT therapist-led intervention for MUPS significantly improved patient-rated depression and somatic symptoms, and clinician-rated severity of symptoms, immediately post-intervention. However, only improvements to patient-rated somatic symptoms were maintained 6-months post-intervention. While depressive and somatic symptom improvements were anticipated as CBT is widely advocated for depression, the improvements in both patient- and clinician-rated MUPS symptoms potentially indicate additional benefits for short-term perceived behavioural and cognitive control. Despite positive results, achieving patient buy-in was problematic as only 41% of eligible patients enrolled with an attrition rate of 45%. While the justification for this was not discussed, the study proposed that future programmes may benefit from using a staged-approach to tailor the intervention to patients’ needs, use of other services, costs, and the delivery setting. As MUPS patients do not benefit from reassurance alone [97] and a similar 6-week CBT programme for Breast Cancer patients demonstrated non-significant results [98], this highlights the need for at least moderate-length, tailored CBT-based interventions that are tailored to patients’ needs. Therefore, while research is required to overcome the confounds of participation and long-term effect maintenance, and to determine how to feasibly implement the complex and time-consuming intervention in practice, CBT demonstrated promise for improving QOL in LTC.

#### Six-month BWM/PCST for knee osteoarthritis

Somers et al.’s [86] clinical psychologist-led 24-week combined PCST and BWM intervention demonstrated significant improvements 12-months post-intervention for the QOL components of arthritis- and weight-specific self-efficacy, pain symptoms and catastrophizing, physical disability and stiffness, weight, and BMI compared to UCC. Additionally, the combined intervention was significantly more effective than the individual interventions for the aforementioned outcomes; excluding PCST for pain catastrophizing and one pain measure. This demonstrates that by conducting a programme which not only targets LTCs’ physical components, but also enables people to cope with the psychological effects and consequences, significantly improves both physical and psychological QOL. However, despite being one of only two studies reviewed to receive a ‘*Strong*’ quality rating, the study was confounded by the combined condition receiving double the intervention dosage than individual conditions. Additionally, as interventions were facilitated by highly trained clinical psychologists, additional research and a cost-benefit analysis comparing this approach with training existing staff involved in arthritis healthcare to provide the intervention would be beneficial. Therefore, while research for potential dose and expertise effects is required, the study demonstrated efficacy for a medium-term intervention to improve QOL 12-months post-intervention through targeting both the physical and psychological components of LTC.

#### Overall medium-term interventions

Overall, the medium-term studies [84, 86] demonstrated effectiveness for interventions delivered by psychologically trained staff to improve QOL in LTC, with CBT resulting in short-term improvements and a combined physical and psychological intervention resulting in improvements 12-months post-intervention. While these studies highlighted the need for medium-term psychological interventions to be tailored to LTC patients’ physical and psychological needs in order to actively involve patients in their healthcare, three considerations are required. First, differences were present in the quality of studies, with Escobar et al. [84] receiving a ‘*Moderate*’ quality rating and Somers et al. [86] a ‘*Strong*’ rating. As this stemmed purely from the CBT-therapist intervention experiencing more problematic ‘*Withdrawals & Dropouts*’ [84], future research into the mechanisms behind this difference would be beneficial. Second, despite both LTC having profound physical and psychological consequences, current understanding of the causes and consequences of MUPS is less well defined than for knee osteoarthritis, which may have impacted outcomes [84, 86]. Third, while the positive outcomes provide an important foundation for research to build upon, consideration is required for the level of staff input and training required to conduct such programmes. As becoming a chartered psychologist or CBT therapist typically takes at least 6–7 years of study and training in addition to vocational work, both programmes required highly specialised staff. While this appears beneficial for QOL outcomes, this raises potential practicality issues for healthcare implementation as considerations would be required to determine capacity, practicality and financial viability within existing or additional services. However, as Somers et al. [86] demonstrated greater improvements based on psychological intervention dosage, this highlights a potential opportunity to utilise psychological principles to improve QOL outcomes for LTC. Therefore, careful consideration is required for the implementation of medium-term interventions using psychologically trained staff; however, the positive effects for both physical and psychological QOL indicate promise for healthcare.

### Long-term interventions (≥12 months)

#### Twelve-month psycho-education and management for HIV

Blank et al.’s [83] 12-month, nurse-led community-based psycho-education and healthcare management intervention for HIV patients demonstrated significant improvements for mental health QOL and immune functioning 12-months post-intervention. However, no effect was present for physical health QOL or viral load. The rationale behind the study was that reforms to healthcare provide a challenge but also an opportunity to redesign systems in a more integrated manner. Through training nurses to facilitate psycho-education while providing tailored access to relevant professions within a multi-disciplinary healthcare team, significant improvements were present for condition-related immune functioning and mental health. However, future healthcare research would benefit from factoring in key confounds. First, as university-based nurses facilitated the intervention the additional research experience associated with this work setting may have influenced outcomes. Second, as viral load changes only occurred 12-months post-intervention, consideration of optimal intervention and assessment duration is required. Finally, while assessing different constructs at different time points may be the most feasible approach within multi-disciplinary interventions, careful consideration is required for the effect this may have on analyses and attrition, as 75% of patients completed the QOL measure 12-months post-intervention compared with only 61% providing bio-markers data. Therefore, while future work may benefit from overcoming practical confounds, altering existing services to provide psycho-education and tailored management of a multi-disciplinary team by nurses may be a feasible, cost-effective approach.

#### Twelve-month counselling for HNC

Van Der Meulen et al.’s [87] 12-month, nurse-led problem-focussed counselling programme significantly improved depressive and physical symptoms in HNC patients immediately post-intervention, with effects being more pronounced in the depressive-subgroup. As the authors proposed that those with greatest physical impairments were more likely to experience depressive symptoms and those with depressive symptoms benefited most from the intervention, problem-focussed counselling demonstrated efficacy both for the general sample and for those patients in greatest need. While the study was confounded by a ‘*Moderate*’ ‘*Selection Bias*’ with only 63% of eligible patients participating, it was one of only two studies to receive a ‘*Strong*’ overall rating and once enrolled attrition rates were low (13%). Therefore, as low attrition supports the authors’ claim that utilising nurse facilitators may not only reduce healthcare costs but also stigma, the intervention was feasible and cost-effective. Hence, due to the positive intervention effects a combined with the psychological elements of the interventions not being specific to HNC, theory-based long-term nurse-facilitated interventions provide promise for LTC healthcare delivery.

#### Overall long-term interventions

Overall, the long-term studies [83, 87] demonstrated efficacy for long-term nurse-led interventions to improve QOL in LTC, with HNC counselling having significant post-intervention effects, and HIV psycho-education and care management improving QOL 12-months post-intervention. Despite differences in the format, content and delivery of interventions, significant QOL improvements were achieved through supporting nurses to facilitate interventions that enabled patients to develop the skills, knowledge and efficacy required to manage the physical and psychological components and consequences of their LTC. Furthermore, as both HIV and HNC are complex LTC that may have profound physical and mental effects and therefore require a large amount of medical support, the positive intervention effects provide promise for other complex LTC. As proposed by Van Der Meulen et al. [87], utilising nurses to provide long-term interventions may be both a financially and practically viable approach to implementing long-term psychological interventions, and may reduce stigma due to nurses already being intrinsically involved in LTC healthcare provision. However, consideration is require for the differences between Blank et al.’s [83] ‘*Moderate*’ and Van Der Meulen et al.’s [87] ‘*Strong*’ quality ratings, with this stemming from the ‘*Weak*' and ‘*Strong*’ ‘*Withdrawals & Dropouts*’ quality ratings respectively. Therefore, future research is required into the mechanisms behind between-study differences in enrolment and attrition despite both interventions utilizing nurse facilitators. Hence, long-term, nurse-led interventions which actively involve patients in their care and target both the physical and psychological constructs of LTC provide promise for healthcare. However, further research is required to determine the optimal approach to adopt in order to enhance patient enrolment for such programmes.

### General discussion

#### Implications of Findings

The studies reviewed demonstrated that psychological interventions for LTC varied considerably in terms of duration, population, methods, quality ratings, facilitators and long-term effectiveness. Descriptive analysis of findings indicated that all interventions resulted in significant improvements to at least one component of QOL immediately-post intervention. Furthermore, the 6-week health educator self-regulation intervention for asthma [82], 6-month clinical psychologist-led combined PCST-BWM intervention for knee osteoarthritis [86], and 12-month nurse-led psycho-education and care management intervention for HIV [83] significantly improved QOL 12-months post-intervention. While further research is required to assess the mechanisms behind differences in the effectiveness of interventions and the feasibility of implementing interventions in LTC healthcare, the findings indicate that psychological interventions utilising different formats, durations and facilitators which actively involve and enable patients to have self-efficacy over their care may result in significant QOL improvements for LTC patients.

In addition to the effectiveness of interventions, the studies have important implications for future research and healthcare. First, across studies enrolment in psychological interventions was low, with one study only successfully enrolling 41% of potential patients [84]. While blinding was often used to increase methodological quality, this may have influenced participation rates through blinding patients to the potential components, goals and benefits of interventions. Additionally, at present LTC treatments typically promote pharmacological and/or medical treatments, with psychological interventions promoted as secondarily [1–3, 5–7, 11, 19, 22, 23, 26]. This may promote patients to seek quick-fix treatments and requires a change in approach in order to enhance participation in psychological interventions. Further, as only 6 RCTs from 2006 to February2016 were deemed suitable based on the inclusion/exclusion criteria, coupled with the review demonstrating that psychological interventions may improve QOL across LTC, this review highlights the need for high-quality research into this area and the application of methods in healthcare. Hence, future research and interventions across LTC that attempt to build upon the positive findings and resolve methodological confounds is recommended in order to build a greater evidence base for the effectiveness of psychological interventions on LTC.

#### General Strengths and Limitations

Many of the strengths of the review may also be regarded as limitations. First, an *ante hoc* decision was made to include only RCTs with a UCC in order to ensure that only high methodological quality studies were included and valid comparisons could be made between interventions despite considerable differences in the LTC targeted [67]. Furthermore, in order to ensure that only the most up-to-date research was assessed, only studies spanning the previous 10 years (2006 to February 2016) were included. While discussions were conducted with relevant experts (within Public Health, Health Psychology and Publishing) prior to the review to set a strict inclusion/exclusion criterion for only the most relevant research, it is possible that important and interesting studies, findings and interventions may have been excluded. Additionally, in order to improve the reliability of findings, only studies that directly targeted LTC patients for both the intervention and assessment were included. However, this may also have reduced the number of interventions through excluding those that indirectly target or assess patients through clinicians, carers or family members, such as communicative or learning disorder populations who may benefit from psychological interventions but are unable to communicate effects. Finally, while he COCHRANE data extraction framework is well validated and used across disciplines [73], the EPHPP quality assessment tool was used as the review aimed to guide public health policy [74]. However, as the review assessed psychological interventions, alternative tools may potentially have been more appropriate and may have resulted in different quality ratings. For example, Smeulders et al. [85] received a ‘*Weak*’ rating despite demonstrating four ‘*Strong*’ components, and Van Der Meulen et al. [87] received a ‘*Strong*’ rating despite only stating significance values as ‘*p ≤ 0.05*’. Therefore, future replications and expansions should attempt to build upon the strengths, and generate solutions for the limitations, of the review in order to improve upon the quality of the review.

#### Rapid systematic review strengths and limitations

Previous research has discussed the relative strengths and limitations of the rapid SR approach compared to traditional SRs [70–72]. One primary benefit of this methodology is that it may be used to assess research and formulate conclusions that influence healthcare policy within a time-frame and budget that would not be possible using traditional methods. While significant work was subsequently conducted to improve the review to publication standard, this methodology allowed the review to progress from defining potential search parameters to providing a first draft to healthcare stakeholders within three-months. Rapid SRs may potentially suffer from using a non-iterative search strategy, narrow time-frame for retrieval, not performing quality analysis, and limiting consultation with experts. However, the present review did not suffer from these confounds as a strict *ante hoc* criteria was set and adhered to, and various contacts (Public Health, Health Psychology etc.) were sought out to discuss the suitability of the review. Therefore, active efforts were made to strengthen methodology by ensuring that many potential confounds of rapid SRs were accounted for.

Despite attempts to maintain as high quality methodology as possible, implicit limitations are associated with one researcher being involved until data extraction. First, practical constraints meant that grey literature, reference lists and additional databases were not searched, which may have provided additional findings. Furthermore, while all possible effort were made to maintain accuracy, ‘*human error*’ and ‘*selection bias*’ are possible, and as only articles published in English were included ‘*publication*’ and ‘*language*’ biases are also possible. However, given the relative strengths and weaknesses of rapid SRs, and that the review was completed during NHS employment (*See Authors’ Information*), overall utilising rapid SR methodology was useful for an initial study. Therefore, future attempts should be made to replicate and expand upon the findings using a larger research team to limit the aforementioned confounds through continuing to utilise a strict *ante hoc* criteria.

## Conclusions

The studies reviewed demonstrated promising results for utilising psychological interventions to improve QOL in LTC patients, with short-, medium- and long-term interventions that promote patient involvement demonstrating positive outcomes. While confounds were present which require resolution, particularly with low participation from eligible patients, the positive results indicated that with high-quality methodology, actively involving patients in their care and tailoring of interventions to patients’ needs, psychological interventions may improve QOL in LTC. Hence, future studies should assess the efficacy of tailored interventions utilising different formats, durations, and facilitators to improve QOL in LTC, while the development and promotion of services should be promoted to utilise psychological interventions to supplement medical care,

## Abbreviations

BWM: Behavioural Weight Management.
CBT: Cognitive Behavioural Therapy
CHF: Congestive Heart Failure
EPHPP: Effective Public Health Practice Project
HIV: Human Immunodeficiency Virus
HNC: Head & Neck Cancer
HR-QOL: Health-Related Quality of Life
LTC: Long-Term Conditions
MUPS: Medically Unexplained Physical Symptoms
MWB: Mental Wellbeing
PCST: Pain Coping Skills Training
QOL: Quality of Life
RCT: Randomised Controlled Trial
SR: Systematic Review
UCC: Usual Care Control
